# Innovative Implementation of an Alternative Tetrathiafulvene Derivative for Flexible Indium Phthalocyanine Chloride-Based Solar Cells

**DOI:** 10.3390/mi12060633

**Published:** 2021-05-29

**Authors:** Leon Hamui, María Elena Sánchez-Vergara

**Affiliations:** Facultad de Ingeniería, Universidad Anáhuac México, Avenida Universidad Anáhuac 46, Col. Lomas Anáhuac, Huixquilucan 52786, Estado de México, Mexico; elena.sanchez@anahuac.mx

**Keywords:** organic semiconductor, thin film, tauc band, solar cells, opto-electrical properties

## Abstract

Herein, we present the photovoltaic properties of an indium phthalocyanine chloride (InClPc)-based flexible planar heterojunction device, introducing the tetrathiafulvene derivative 4,4′-Dimethyl-5,5′-diphenyltetrathiafulvalene (DMDP-TTF) as the electron donor layer. UV-vis spectroscopy is widely used to characterize the electronic behavior of the InClPc/DMDP-TTF active layer. The interactions between the DMDP-TTF and phthalocyanine are predominantly intermolecular and the result of the aggregation of InClPc. Tauc bands were obtained at 1.41 and 2.8 eV; these energy peaks can result in a charge transfer ascribed to the transition from the DMDP-TTF to π-orbitals that are associated with the phthalocyanine ring or even with the same indium metal center. Conductive carbon (CC) was used for the cathode. Finally, an indium tin oxide (ITO)/InClPc/DMDP-TTF/CC device was fabricated by high-vacuum thermal evaporation onto a flexible substrate and the photovoltaic properties were evaluated. A diode type I-V curve behavior was observed with a photovoltaic response under illumination. A generated photocurrent of 2.25 × 10^−2^ A/cm^2^ was measured. A conductivity reduction with the incident photon energy from 1.61 × 10^−7^ S/cm to 1.43 × 10^−7^ S/cm is observed. The diode resistance presents two different behaviors with the applied voltage. A V_TFL_ of 5.39 V, trap concentration of 7.74 × 10^16^ cm^−3^, and carrier mobility values of ~10^−6^ cm^2^/V s were calculated, showing improved characteristics via the innovative implementation of an alternative TTF-derivative, indicating that the DMDP-TTF has a strong interaction at the junction where free available states are increased, thus inducing higher mobilities due to the large number of π-orbitals, which indicates the feasibility of its use in solar cells technology.

## 1. Introduction

In the last decade, several investigations have focused on the development of organic semiconductors for the fabrication of next-generation photovoltaics cells, mainly by their capability to be fabricated on large surface areas and generate lightweight and flexible devices at a low cost. The latter, trying to reduce the production costs, will be rapidly affected by the introduction of new materials. In addition, by molecular design, these materials allow the engineering of their optoelectronic properties, due to their simple synthesis [1,2,3]. Moreover, they allow for an economically and environmentally sustainable life-cycle of the device [4,5]. However, the organic device’s stability is still limited by a few factors such as irradiation, heating and mechanical stress, metastable morphology, among others. Trying to resolve this problem, investigations have shown that stability can be increased by design strategies in which active layer material design and device engineering could also increase the power conversion efficiencies for organic solar cells technology [6,7,8]. Some non-fullerene-based active layer solar cells have shown a predicted 27,000 year lifetime [9,10]. To achieve this, an efficient exciton dissociation and charge carrier transport process, and a reduction in the charge recombination process, should be designed for the active layer material [11]. Still, a challenge due to the limited exciton diffusion length is present, whereby mainly excitons within a short distance from the donor–acceptor interface can be dissociated [12,13]. Some of the organic semiconductors that have attracted attention for optoelectronic device applications are based on molecules that are highly conjugated and display rich π-electrons. Among several compound families, such as small molecules, charge transfer complexes, discotic systems, fullerenes, and compounds formed by naphthalene units (naphtalenes, perylenes, rylenes, etc.), the metal phthalocyanines (MPcs) are comprised of discotic systems, which show interesting optoelectronic properties for solar cells technology [14,15,16,17,18]. In particular, the MPcs possess planarity, symmetry, and π-orbitals that are energetically accessible for charge transport [2,19], where the promising photovoltaic response and rapid electron density delocalization, which occurs throughout the macrocycle, give rise to marked photoconductivity and rapid conduction inside the MPc-based films [19,20,21,22,23]. These are a few of their interesting properties that make them good candidates for electronic devices [20,24,25], and furthermore for solar cells [21,26,27,28,29], where they can be alternately incorporated by doped and undoped MPc layers for heterostructure solar cells [30,31]. Among many MPcs, copper phthalocyanine (CuPc) and zinc phthalocyanine (ZnPc) have been widely studied as potential materials for solar cells due to their appealing photovoltaic and photoconductive properties [32,33,34,35]. Furthermore, indium(III) phthalocyanine chloride (InClPc) Schottky barrier devices have been investigated, which show a promising optoelectronic performance for solar cells and nonlinear optic devices, employing this material as the active layer [24,32,33,34,36]. The metalic atom (In) in the InClPc allows for bonding with the chloride ion, which promotes charge carrier transport due to its high electronegative and polarity. This modification in the metal center coordination sphere is only allowed on several MPcs such as indium Pc. The latter may be an advantage when these types of Pc are used as part of the active layer in optoelectronic devices, unlike the Cu and Zn atoms in their respective MPcs [37]. Few investigations have been conducted on incorporating InClPc as a planar junction solar cell, but a conversion efficiency under an illumination intensity of 100 W/m^2^ of ~3% has been previously observed [32]. The latter is a consequence of good light absorption in the visible region of spectra, where the InClPcs show a molecular extinction coefficient (ε) > 10^5^ dm^3^ M^−1^ cm^−1^ and optical and fundamental energy gaps of 1.51 and 2.9 eV, respectively [35]. The transport properties of InClPc are strongly related to the excitation-quenching process between highest occupied molecular orbital (HOMO) and lowest unoccupied molecular orbital (LUMO). Like most MPcs, InClPcs can be used as an electron acceptor in solar photovoltaic cells, and have been known to undergo electron-transfer reactions with strong electron donors such as tetrathiafulvalene (TTF) [38]. The TTF has the ability to form ordered stacks, and to afford cation radical salts with remarkable electronic properties [39]. TTF and its derivatives have shown an electronic donor behavior, which plays an important charge transport role in electronic devices [39,40]. TTF derivatives have been investigated for electronic and heterojunctions solar cell applications [41,42,43,44], as well as for dye-sensitive solar cells, where a broad absorption spectrum is observed [45]. Another case is organic field-effect transistors (OFETs) based on TTF derivatives, the properties of which are stabilized and improved by the introduction of substituents of TTF [46]; examples are HMTTF (hexamethylene-TTF) [47], OMTTF (octamethylene-TTF) [48], and DBTTF (dibenzo-TTF) [49]. Thus, the use of TTF derivatives with this type of MPc for solar cells, which may allow better charge transport and optoelectronic properties than other donor materials, could solve the issues of a deficient exciton dissociation and charge carrier transport process on the active layer of an organic solar cell. The main aim of the current work is to study the photovoltaic behavior of the active layer formed by the acceptor InClPc and the 4,4′-Dimethyl-5,5′-diphenyltetrathiafulvalene (DMDP-TTF). InClPc and DMDP-TTF are the semiconductor constituents of the active layer that will be responsible for the absorption of radiation, and on which the charge carriers that produce the photocurrent are generated. The DMDP-TTF is a derivative of a TTF that has been very little studied as an electron donor film, and its incorporation could play a key role in solar cell devices. The functionalization of the TTF derivative provides the opportunity to correlate the electronic properties of DMDP-TTF with structural features of InClPc, in order to ultimately be able to control and manipulate the properties of the photovoltaic devices [50]. To study the photovoltaic behavior of the InClPc/DMDP-TTF active layer, a planar heterojunction device will be manufactured and characterized. J-V measurements were conducted under darkness and illuminated conditions to evaluate the photovoltaic behavior and device parameters, such as the ideality factor, carrier mobility, photocurrent and trap concentration, among others. In the case of the formation of these heterostructures with thin films, the interactions at the interfaces should be taken into account, in addition to balancing the interactions between the InClPc and DMDP-TTF molecules, to improve the organic solar cell performance. Finally, it is important to consider that new solar cell applications require the use of flexible substrates; it is for this reason that in the manufacture of the device, polyethylene terephthalate (PET) was used as a substrate. The PET presents better properties compared to other polymeric materials, such as flexibility, high mechanical properties, low-temperature large-area processing, recyclability, and roll-to-roll deposition techniques, which can also be easily covered with high-quality indium tin oxide (ITO) for device fabrication [51,52,53].

## 2. Materials and Methods

**Figure 1:**
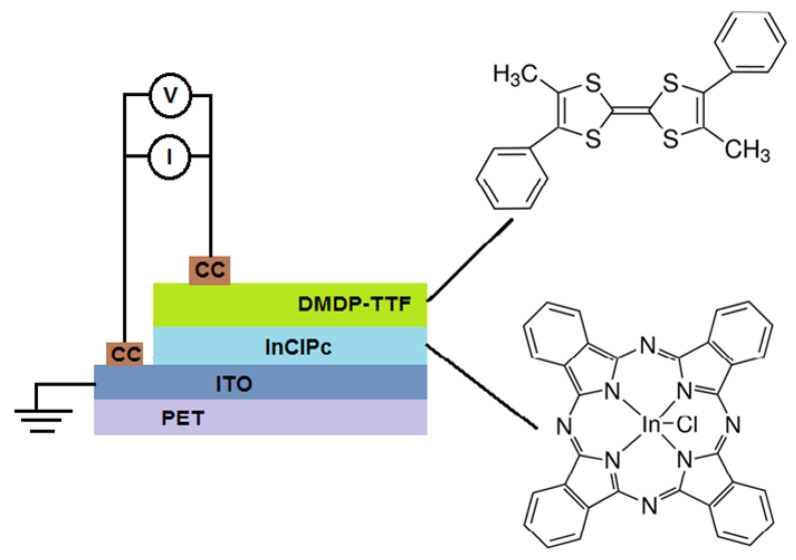
Schematic illustration of polyethylene terephthalate (PET)/indium tin oxide (ITO)/indium phthalocyanine chloride (InClPc)/4,4′-Dimethyl-5,5′-diphenyltetrathiafulvalene (DMDP-TTF)/conductive carbon (CC) device.

The InClPc (Indium(III) phthalocyanine chloride: C_32_H_16_N_8_InCl) and the DMDP-TTF (4,4′-Dimethyl-5,5′-diphenyltetrathiafulvalene: C_20_H_16_S_4_) were obtained from Sigma-Aldrich (Saint Louis, MO, USA) and required no further purification. The InClPc/DMDP-TTF active layer was fabricated using a high-vacuum thermal evaporation system (HVTE, Intercovamex, Morelos, Mexico). A tantalum boat source was used for evaporation and was set to 550 K. The selected pressure in the vacuum chamber was 1 × 10^−5^ torr before film deposition. The evaporation rate was 2 Å/s, thus requiring a film thickness of 92 nm. The latter was measured by a quartz-crystal microbalance monitor connected to a thickness sensor. Commercial indium tin oxide (ITO, In_2_O_3_·(SnO_2_)_x_)-coated polyethylene terephthalate film (ITO-PET, 100 Ω/sq, Sigma-Aldrich, MO, USA), quartz, and monocrystalline silicon substrates were used for sample deposition and further characterization. Quartz substrates were ultrasonically degreased in 1,2-dichloroethane, methanol and acetone. Meanwhile, Si substrates were chemically etched for 5 min with a 10 mL HF, 15 mL HNO_3_ and 300 mL H_2_O solution in order to remove the native oxide from the c-Si surface. For the device manufacturing, the following process was used (see Figure 1): InClPc and DMDP-TTF were deposited into separate boat sources for consecutive deposition, the InClPc was used as the electron acceptor layer and the DMDP-TTF as the electron donor layer, and the ITO was used as the anode while the conductive carbon (CC, Sigma-Aldrich, MO, USA) was used as the cathode and was deposited at the end of the process, in order to improve the charge carrier collection. Fourier-Transform Infrared (FTIR) spectroscopy analysis was carried out on a silicon substrate sample, which helped to verify the formation of chemical bonds and functional groups with a Nicolet iS5-FT spectrometer (Thermo Fisher Scientific Inc., Waltham, MA, USA) on a wavenumber range of 4000 to 500 cm^−1^. UV-vis spectroscopy allowed for measurement of the optical transmittance and absorption in the 200–1100 nm spectral range using a UV-vis spectrophotometer (Thermo Fisher Scientific Inc., Waltham, MA, USA) on a quartz substrate sample. The electrical properties were obtained using a sensing station with a lighting controller circuit from Next Robotix (Comercializadora K Mox, S.A. de C.V., Mexico City, Mexico) and an autoranging Keithley 4200-SCS-PK1 pico-ammeter (Tektronix Inc., Beaverton, OR, USA). A conduction channel of 1 cm × 0.1 cm was selected for the device. The lighting system, with light-emitting diodes (LEDs) as the light source, allowed the irradiation of the samples with 7 light colors: UV (2.70 eV), blue (2.64 eV), white (2.57 eV), green (2.34 eV), yellow (2.14 eV), orange (2.0 eV), and red (1.77 eV).

## 3. Results and Discussion

### 3.1. Deposit and Characterization of Semiconductor Films

**Figure 2:**
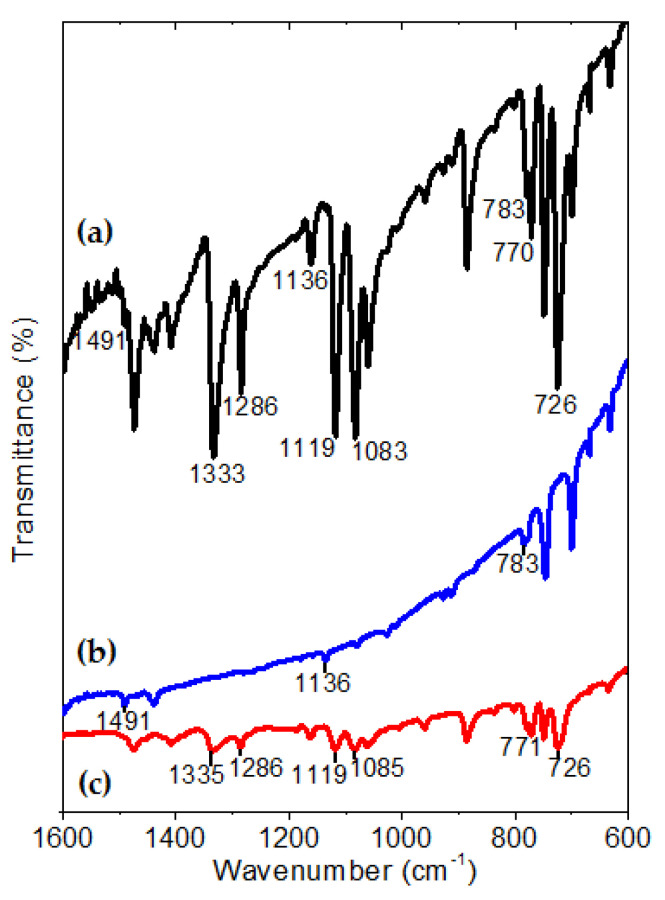
Infrared absorption spectra of (**a**) InClPc/DMDP-TTF films, (**b**) DMDP-TTF and (**c**) InClPc in KBr pellets.

After the deposit, FTIR spectroscopy was carried out to identify the principal chemical bonds and functional groups in the InClPc and DMDP-TTF films. The deposition of thin films by the HVTE method resulted in phase changes in the organic semiconductors. These physical changes in the material can induce degradation of the molecular skeleton of InClPc and DMDP-TTF. FTIR spectroscopy helps to determine whether or not the materials underwent a chemical decomposition. The FTIR spectrum of the InClPc/DMDP-TTF films was compared with the spectra of the InClPc and DMDP-TTF compounds, and Figure 2 shows the most important signals. The InClPc spectrum is characterized by out-of-plane C-H mode (726 cm^−1^), in-plane C-H bending modes (1085, 1119, and 1286 cm^−1^) and in-plane C-N mode (1332 cm^−1^) [37,54,55,56]. On the other hand, the DMDP-TTF spectrum is characterized by in-plane C-S mode (1491 cm^−1^), in-plane C-H mode (1136 cm^−1^) and out-of-plane C=C mode (783 cm^−1^) [57]. According to the spectrum in Figure 2a, the deposited InClPc/DMDP-TTF films were formed by the same functional groups as those belonging to the DMDP-TTF and InClPc. Small variations in the signals of 1083 and 1333 cm^−1^ for in-plane C-H and C-N modes, respectively, and referring to phthalocyanine, were generated by the concentration of stress during the deposition of the films. However, these results indicated that the HVTE method did not chemically degrade the InClPc or the DMDP-TTF, and can be used for the preparation of films. On the other hand, phthalocyanines such as InClPc present in thin films a certain crystalline form, or become amorphous depending on the film fabrication procedure [37]. Information about the structure and molecular orientation can be extracted by comparing the intensity of the vibrational modes of InClPc KBr pellet and film spectra [37]. The FTIR spectrum of the InClPc/DMDP-TTF films is dominated by the strong intensities of the in-plane mode signals of the phthalocyanine. These signals, where the in-plane modes in the FTIR spectrum are clearly enhanced, correlate with a preferential orientation of the InClPc molecules on the surface of the film. Additionally, the FTIR spectrum is used to identify the phase nature of InClPc, as the spectrum is markedly dependent on the chemical composition and its crystalline form [56]. MPcs are known to have different polyforms, which can be identified by FTIR spectroscopy [58,59]. Two polymorphs (α-metastable and β-stable) can occur because of the slight differences in the π–π electronic interaction between molecules in close proximity within the lattice [56]. The β-form gives rise to vibrations at about 770 cm^−1^, while the α-form produces vibrations at about 720 cm^−1^ [58,60]. Figure 2 shows the infrared absorption spectra, where the two crystalline forms are shown with the values of 723 and 768 cm^−1^, respectively, for α- and β-form. The presence of both crystalline forms may affect the charge transport and conduction properties of the device, due to the electronic interaction within the InClPc and at the InClPc/DMDP-TTF junction.

**Figure 3:**
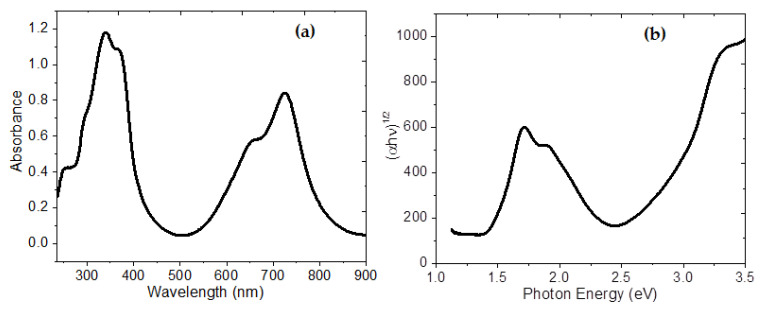
(**a**) UV-vis spectrum and (**b**) Tauc Plot of InClPc/DMDP-TTF active layer.

The active layer was fabricated by the sequential deposition of InClPc and DMDP-TTF films. UV-vis spectroscopy is widely used to characterize the electronic properties of the InClPc/DMDP-TTF active layer through the determination of parameters describing the electronic transitions, such as band gap [61]. The UV-vis spectra of the InClPc/DMDP-TTF active layer deposited on quartz are shown in Figure 3a. The region between 250 and 400 nm in the spectrum shows superimposed bands of InClPc and DMDP-TTF species [62,63]: (i) the absorption around 255 nm is assigned to the DMDP-TTF film and (ii) the InClPc film shows a Soret electronic absorption band with two peaks at 340 and 380 nm [37,54,55,56,57,58,59,60,64,65]. The Soret band indicates the presence of a d-band associated with the central metal atom in InClPc [65,66]. In addition to the Soret band in the near ultraviolet region, the InClPc has a band in the visible region called the Q band [37,54,55,56,57,58,59,60,64,65]. The Q band has two peaks at wavelengths of 650 and 724 nm, and the high energy peak of the Q band has been assigned to the first π→π* transition on the phthalocyanine macrocycle. The low energy peak of the Q band has been explained variously, as a second π→π* transition, as an exciton peak, as a vibrational internal interval and as a surface state [60]. The characteristic splitting in the Q band is typically observed in the α-form in the InClPc films [37]. The UV-vis characterization suggests that the HVTE method for phthalocyanine powders leads to the formation of films where the α-form is predominant.

The band gap is the important parameter in the physics of semiconductors, and was determined from the optical absorption coefficient (α) and photon energy (hν) [67]. The Tauc band gap associated with the InClPc/DMDP-TTF active layer is determined through an extrapolation of the linear trend observed in the spectral dependence of (αhν)*^n^* over a range of hν [67]. For the allowed indirect transitions, *n* = 2, h is Planck’s constant and ν is the frequency. As shown in Figure 3b, two band gaps were obtained at 1.41 and 2.8 eV; the first refers to the presence of InClPc, and the second refers to DMDP-TTF. These energy peaks can result in a charge transfer ascribed to the transition from the donor DMDP-TTF to π-orbitals that are associated with the acceptor phthalocyanine or even with the same indium metal center. In active layers formed by planar heterojunction, charge transport is generated at the donor–acceptor interface. The preferential orientation of the InClPc molecules on the surface of the film favors the transport of charges from the DMDP-TTF.

### 3.2. Manufacture and Characterization of the Device

**Figure 4:**
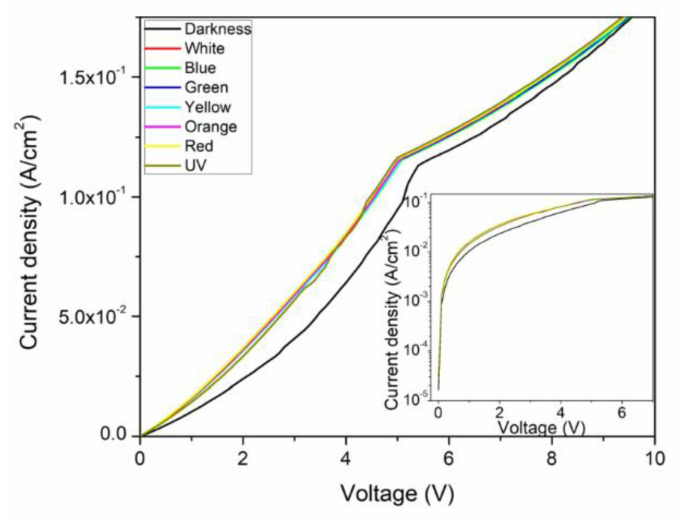
J-V characteristics and J-V semilogarithmic inset under dark and various illuminated conditions.

For electrical characterization, J-V measurements were conducted under darkness and illuminated conditions. On Figure 4, we can see a diode-type characteristic with a two-step shape for all curves. The darkness condition characteristic has lower current density values than those of the illuminated conditions, therefore indicating a photovoltaic response under different light wavelengths. The observed photovoltaic response is more pronounced for voltages lower than 6 V. For the darkness condition, a 5.0 × 10^−2^ A/cm^2^ current density value can be observed at 3.50 V, whereas for the illuminated condition a 7.50 × 10^−2^ A/cm^2^ current density value can be observed at 3.50 V. Nevertheless, this variation is diminished for voltage values close to 9 V, where it tends to 1.70 × 10^−1^ A/cm^2^. The latter observations can be corroborated on the Figure 4 inset, where the semilogarithmic J-V curve is shown. The increase in current is due to an increase in the photogenerated carrier’s concentration, a consequence of the active layer’s interaction with the incident light where absorption take place, resulting from the incident photon energy being larger than the band gap, which allows the electronic interaction of the donor–acceptor system, which is subsequently followed by the exciton’s dissociation at the interface [68]. Additionally, there is a slight variation in the curve depending on the incident light wavelength, observed on Figure 4. It is important to point out that the molecular extinction coefficient of the InClPc varies with wavelength [35], resulting in a change in photon absorption, which may contribute to the charge transport, and then current generation, which impacts the device’s external quantum efficiency (EQE) [68]. The previous, combined with the exciton diffusion length and absorption depth, which may favor certain light wavelengths, support the observed current density variation with the incident light.

**Figure 5:**
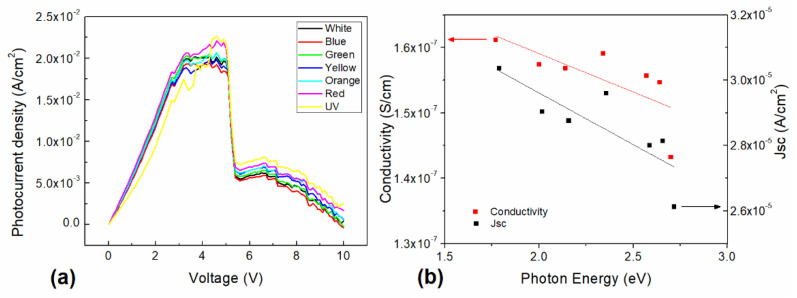
(**a**) Photocurrent density vs. voltage characteristic for various illuminated conditions. (**b**) Device conductivity and Jsc as a function of the photon energy.

Moreover, a deeper analysis was conducted to evaluate the photovoltaic effect, and the generated photocurrent density characteristics under different illumination wavelengths (seven light colors) were plotted (Figure 5a). First, it can be observed that all photocurrent density characteristics increase and then tend to a maximum constant value as they approach 5 V. The highest photocurrent value observed in Figure 5a is of 2.25 × 10^−2^ A/cm^2^ (for the UV light, 2.7 eV), while the lowest maxima observed is of 1.90 × 10^−2^ A/cm^2^ (for the blue light, 2.64 eV). Moreover, for a 5 V fixed value, an average increment of ~22% of the current density is observed, compared to the darkness condition. Second, a steep decrease in the photocurrent density (1.31 × 10^−2^ A/cm^2^) is observed at 5.50 V, a change of 65% on the photocurrent density, which may indicate that the recombination rate is increased, as well as a saturation of photogenerated carriers within the depletion region. Finally, a decay of the generated photocurrent density is observed for voltage values higher than 7 V, of 0.50 × 10^−2^ A/cm^2^. This may be related to the rapid recombination, as the increase in the bias voltage shortens the gap between the HOMO and LUMO of the two materials and the probable full trap filling state, which affects the conduction mechanism for the photogenerated carriers. Additionally, it is important to consider that the HOMO–LUMO gap or band gap for InClPc is 2.2 eV [69], while the band gap for TTF-derivatives is around 3.65 eV [46]; nevertheless, for the InClPc /DMDP-TTF active layer, the Tauc band gap values obtained are 1.41 and 2.80 eV. These results indicate an increase in charge transport within the same molecule and between different molecules. It is important to note that the photocurrent density values at 10 V are between 0 and 0.25 × 10^−2^ A/cm^2^, depending on the incident light color, although most of the curves present a similar shape for voltages lower than 3 V, except for the UV light curve, which presents lower photocurrent density values. The latter may be related to the observed optical gap, where a wavelength close to the 1.41 eV bandgap achieves more efficient carrier generation and collection, as opposed to the 2.80 eV bandgap, where the photogenerated carriers in the DMDP-TTF increase the recombination of the transported low-minority carriers generated in the InClPc. However, while the rest of the curves are followed by a constant photocurrent density value, the red light and UV light curves keep increasing, but the UV light curve became the highest. It is interesting to note that for higher voltages, the photocurrent density presents a distinct behavior for each of the incident light colors, showing an increase with light colors as follows: 2.64 < 2.57 < 2.34 < 2.14 < 2.0 < 1.77 < 2.7 eV. The latter could be related to the resulting observations of UV-vis spectroscopy, where phtalocyanine macrocycle π→π* transitions are preferred, as the photon energy decreases for the selected light source wavelengths. Thus, higher voltages allow a different conduction mechanism, which enhances the carrier collection at the contacts, diminishing the previously observed effect of lower voltages. Figure 5b shows the device conductivity and short circuit current density (Jsc) as a function of the photon energy. The conductivity shows a slight decrease with the incident photon energy, from 1.61 × 10^−7^ S/cm to 1.43 × 10^−7^ S/cm, with a resulting slope of 1.17 × 10^−8^. On the other hand, the Jsc also presents a decrease with the incident photon energy. The observed decrease is from 3.04 × 10^−5^ A/cm^2^ to 2.61 × 10^−5^ A/cm^2^, with a resulting slope of 3.16 × 10^−6^. Furthermore, for the 2.7 eV light, the observed decrease is more marked. Nevertheless, in comparison to the darkness conductivity, where 1.03 × 10^−7^ S/cm conductivity and 1.64 × 10^−5^ A/cm^2^ Jsc were observed, the device presents an increase under illumination conditions. A conductivity increase of 56% and a Jsc increase of 85% were observed between both states (compared to the lowest photon energy). However, all the illuminated conductivity and Jsc values are higher than those under darkness conditions. Nevertheless, these results support the previous observations, wherein the device’s illuminated-state conductivity and Jsc are affected by the optical absorption of both materials within the planar heterojunction structure.

**Figure 6:**
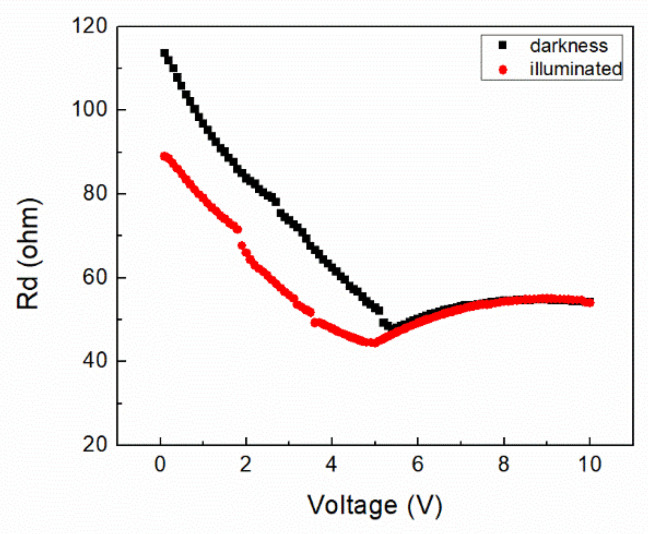
Diode resistance vs. applied voltage.

The diode resistance (Rd) is plotted as a function of the applied voltage on Figure 6, for darkness and illuminated conditions. The diode resistance is lower for the illuminated condition than for the darkness condition due to an increase in the charge carrier density, a result of the photogenerated exciton dissociation at the InClPc/DMDP-TTF junction. Additionally, the Rd is inversely proportional to the device current, which is increased under the illumination condition, ands this results in a diminution of the Rd. We can observe two different behaviors; the first one is a decrease in the device’s resistance with the applied voltage, from 115 Ω to 55 Ω, and the second is the constant device resistance (~55 Ω) with applied voltage for voltages higher than 5.5 V. The lowest device resistance was observed at 5.50 V with 50 Ω. It can be observed that the device resistance is reduced under illumination, and the decrease occurs at 0 V from 115 Ω to 90 Ω, which is an almost 22% reduction. However, for the illuminated condition, the reduction in the device’s resistance with applied voltage is from 90 Ω to 45 Ω. This is followed by a curve behavior of similar values to the darkness condition.

Further device analysis was conducted, and diode parameters are included in Table 1. Due to ohmic contact between the electrodes and the organic semiconductor film, the current density for the space charge limited current (SCLC) region can be expressed as follows:

(1) $$J_{\mathrm{SCLC}}=\frac{9\varepsilon_{r}\varepsilon_{0}{\mu V}^{2}}{8L^{3}}$$

where J is the current density, μ is the mobility, V is the applied voltage, L is the film thickness, and $\varepsilon_{r}$ and $\varepsilon_{0}$ are the relative material (3.6 for the CuPc [70]) and vacuum (8.85 × 10^−14^ Fcm^−1^) permittivity, respectively.

Moreover, the device’s J-V curve can be divided into three conduction mechanism regions: OHMIC, T-CLC (trap-charge limited current) and SCLC (space charge limited current). Each of the conduction regions slopes are shown on Table 1, which suggests that there are different conduction mechanisms that control the charge transport. For the ohmic region, the obtained value is close to 1, which indicates the linear behavior of the current density, whereas for the T-CLC and SCLC regions the slope varies and is more pronounced for the T-CLC region, indicating that the device conduction mechanism is dominated by traps. However, the SCLC value should be approximately 2, but the obtained value was noticeably lower than that, suggesting that the charge mobility within the device was affected. Among the parameters shown on Table 1, the trapping factor, photocurrent, trap concentration, V_TFL_, Jsc, ideality factor, and barrier height (φb) are observed. The current density in TCLC can be expressed as [70]:

(2) $$J_{\mathrm{TCLC}}=\frac{9\varepsilon_{r}{\theta \varepsilon }_{0}{\mu V}^{2}}{8L^{3}}$$

where θ is the trapping factor, which is the ratio of free to trapped charges. The trap filling limit voltage (V_TFL_) is where all the traps are filled, and the current density increases abruptly, but beyond this point remains in the SCLC region. Therefore, the V_TFL_ is related to the material’s intrinsic concentration of traps (N_t_) by the following expression:

(3) $$N_{t}≅\frac{\varepsilon_{r}\varepsilon_{0}V_{\mathrm{TFL}}}{{e\;L}^{2}}$$

A trap filling limit voltage (V_TFL_) of 5.39 V is reported in Table 1. This result supports the previous photocurrent and diode resistance observations. The calculated trap concentration was of 7.74 × 10^16^ cm^−3^, and the trapping factor was 3.48, which is quite large compared to previous reports [71,72]. Such trap concentrations are in accordance with previous works (10^16^~10^18^ cm^−3^) [73,74]. However, the free charges concentration, related to the density of states, is of greater value than the trap concentration. Hence, these observations may indicate that DMDP-TTF strongly interacts at the junction where free available states are increased, due to the large number of π-orbitals, where higher mobilities could be induced.

The carrier mobility was calculated using Equation (1) and by fitting the slope of the J-V^2^ curve on the SCLC region; therefore, the current was only carrier mobility-dependent. Table 1 contains the estimated mobility value for the device in the SCLC region, where it can be observed that the obtained mobility values (~10^−6^ cm^2^/V s) are of similar order of magnitude to those reported for TTF derivatives with phenyl substituents (10^−6^–10^−5^ cm^2^/V s) [75] or MPcs such as ZnPc (10^−6^ cm^2^/V s) [76]. The improved mobility is a consequence of the intramolecular charge interaction between the DMDP-TTF and the InClPc for the planar heterojunction. Also, due to the relatively strong electric field generated at the junction, but it is also important to consider the effect of each of the molecules that make up the active layer. The chloride in the fifth position of the coordination sphere of indium (III) in the phthalocyanine increases the polarity in the molecule and its capacity as an electron-attractor. On the other hand, the molecules of DMDP-TTF are almost planar, and the phenyl groups are tilted 5° and 9° from the TTF plane [75]. This gives rise to preferential directions of charge transport along the phenyl substitutes. The forward current through the Schottky contacts was determined using the following expression:

(4) $$I=I_{s}\exp\left(\frac{\mathrm{qV}}{\mathrm{nkT}}\right)$$

where V is the applied voltage, I_s_ is the saturation current, and n is the diode ideality factor. The saturation current was determined in the reverse bias using the following expression:

(5) $$I_{s}={\mathrm{AA}}^{*}\exp\left(\frac{-{q\varphi }_{b}}{\mathrm{kT}}\right)$$

where A is the diode field, A* is the effective Richardson constant (1.3 × 10^5^ A/cm^2^ K^2^ for the ZnPc [77]), T is the absolute temperature, q is the electron charge, φb is the barrier height, and k is the Boltzman constant (8.62 × 10^−5^ eV/K). The ideality factor can be calculated by the ln(J)-V linear region slope. Moreover, the barrier height can be calculated from the intersection of the line with the current axis and derived from the following expression:

(6) $$\varphi_{b}=\frac{\mathrm{kT}}{q}\left(\frac{{\mathrm{AA}}^{*}T^{2}}{I_{s}}\right)$$

The photocurrent density shown on Table 1 is at 0 V, which is observed to be of 1.17 × 10^−5^ A/cm^2^. The latter indicates the purely incident light effect on the generation of carriers that were collected. On the other hand, the barrier height energy was calculated from Equation (6), and was 1.12 eV, which, compared to previous reports for this type of material, is quite high (NiPc ~0.96 eV, CoPc ~1.02 eV) [77,78].

**Figure 7:**
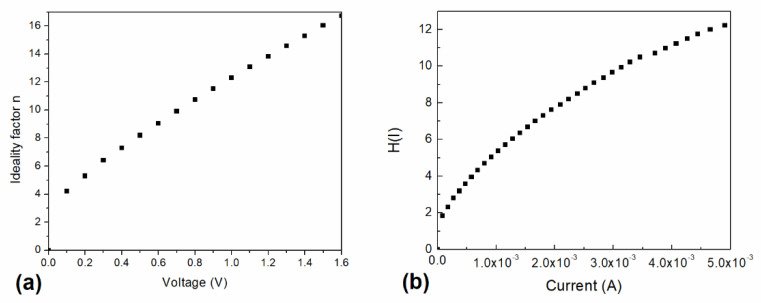
(**a**) Voltage-dependent device ideality factor. (**b**) H(I) vs. current.

The calculated ideality factor derived from Equation (4) is 0.97, which approximates and is in accordance with the ideal diode. However, the voltage-dependent ideality factor (*n*) of the device is shown in Figure 7a for voltages ranging between 0 and 1.6 V. The n values are found to be relatively higher than the ideal diode (*n* = 1) for voltages higher than 0.1 V. The values are found to be between 4 and 17, indicating that the diode presents a nonideal behavior as a possible consequence of the lack of film homogeneity and the interfaces. Additionally, the observed changes in the ideality factor may indicate that the device suffers from high electron hole recombination in the depletion region. A slight change in the slope is observed, which may be related to the effect of the device resistance, dominated by shunt and series resistance for lower and higher voltages, respectively. Therefore, the observed effect on the device’s resistance may be caused by an increase in the series resistance to a saturation value, which causes the current density through the device to present an almost direct relation with the applied voltage.

Cheung and Cheung defined the following expression [79]:

(7) $$H\left(I\right)={n\varphi }_{b}+R_{s}I$$

Figure 7b shows the current-dependent H(I) for the InClPc/DMDP-TTF device. A slope and intercept with the y-axis of 3.81 × 10^3^ and 1.67, respectively, are observed. The intercept is related to the barrier height, which is in agreement with the previously obtained value (Table 1). It is interesting to notice that a device H(I) value of 5.22 was obtained with a current of 1 × 10^−3^ A.

## 4. Conclusions

The photovoltaic behavior of an active layer integrating InClPc and DMDP-TTF, used as acceptor and electronic donor species, respectively, was evaluated. The DMDP-TTF was incorporated as an innovative TTF-derivative for MPc organic solar cells. The InClPc/DMDP-TTF layer was optically characterized via its absorbance and its optical gap. In addition to the Q and B-band characteristics related to InClPc, two band gaps were obtained at 1.41 and 2.8 eV. The ITO/InClPc/DMDP-TTF/CC device was fabricated and electrically characterized. Electrical characterization was conducted under darkness and illuminated conditions. A diode-type characteristic with a two-step shape can be observed with a photovoltaic response under different light wavelengths. A photocurrent of ~2.25 × 10^−2^ A/cm^2^ (for the UV light, 2.7 eV) and of 1.90 × 10^−2^ A/cm^2^ (for the blue light) can be observed. The current density at 5 V shows an average increment of ~22% compared to the darkness condition. For voltages higher than 5.50 V, the photocurrent density presents a distinct behavior depending on the incident light color (2.64 < 2.57 < 2.34 < 2.14 < 2.0 < 1.77 < 2.7 eV). The conductivity decreases with the incident photon energy from 1.61 × 10^−7^ S/cm to 1.43 × 10^−7^ S/cm. The illuminated conductivity and Jsc values are higher than those under darkness conditions. The diode resistance presents two different behaviors with the applied voltage: a decrease in the device resistance with an applied voltage from 115 Ω to 55 Ω at 5 V, and a constant device resistance of ~55 Ω for higher voltages. A V_TFL_ of 5.93 V, trap concentration of 7.74 × 10^16^ cm^−3^, trapping factor of 3.48 and carrier mobility value of ~10^−6^ cm^2^/Vs were calculated. The improved mobility is a consequence of the intramolecular charge interaction between the DMDP-TTF and the InClPc at the planar heterojuntion. Thus, the incorporation of the DMDP-TTF allows for a strong interaction at the junction where free available states are increased, inducing higher mobilities due to the large number of π-orbitals, as observed in the previous results. All of the latter indicates that the InClPc/DMDP-TTF device shows interesting photovoltaic properties, with potential applications in solar cells technology. Further work should be conducted to improve the photovoltaic response by device engineering, and to evaluate the organic solar cell’s parameters, such as the device power conversion efficiency. Additionally, EQE measurements should be conducted to correlate the absorption and exciton dissociation processes with the photogenerated current and solar cell parameters.

## Figures and Tables

**Table 1:** Device parameters for the solar cell structure in darkness.

Parameter	Units	Darkness
SLOPE OHMIC	-	1.09
SLOPE T-CLC	-	1.69
SLOPE SCLC	-	0.8
Mobility	cm^2^/V s	3.58 × 10^−6^
Trapping Factor	-	3.48
Photocurrent density @ 0 V	A/cm^2^	1.17 × 10^−5^
Trap concentration	cm^−3^	7.74 × 10^16^
V_TFL_	V	5.39
Jsc	A/cm^2^	1.64 × 10^−5^
Ideality Factor (n)	-	0.97
Barrier Height (φ_b_)	eV	1.12

## Data Availability

Data is contained within the article.
